# SERPINE1: Role in Cholangiocarcinoma Progression and a Therapeutic Target in the Desmoplastic Microenvironment

**DOI:** 10.3390/cells13100796

**Published:** 2024-05-07

**Authors:** Ralf-Peter Czekay, Craig E. Higgins, Hasan Basri Aydin, Rohan Samarakoon, Nusret Bekir Subasi, Stephen P. Higgins, Hwajeong Lee, Paul J. Higgins

**Affiliations:** 1 Department of Regenerative & Cancer Cell Biology, Albany Medical College, Albany, NY 12208, USA; czekayr@amc.edu (R.-P.C.); higginc@amc.edu (C.E.H.); samarar@amc.edu (R.S.); higgins@amc.edu (S.P.H.); 2 Department of Pathology & Laboratory Medicine, Albany Medical College, Albany, NY 12208, USA; aydinh@amc.edu (H.B.A.); subasin@amc.edu (N.B.S.); leeh5@amc.edu (H.L.)

**Keywords:** cancer-associated fibroblasts, cholangiocarcinoma, extracellular matrix, tumor microenvironment, plasminogen activator inhibitor-1, SERPINE1, p53, cell signaling, tumor progression

## Abstract

A heterogenous population of inflammatory elements, other immune and nonimmune cells and cancer-associated fibroblasts (CAFs) are evident in solid malignancies where they coexist with the growing tumor mass. In highly desmoplastic malignancies, CAFs are the prominent mesenchymal cell type in the tumor microenvironment (TME), where their presence and abundance signal a poor prognosis. CAFs play a major role in the progression of various cancers by remodeling the supporting stroma into a dense, fibrotic matrix while secreting factors that promote the maintenance of cancer stem-like characteristics, tumor cell survival, aggressive growth and metastasis and reduced sensitivity to chemotherapeutics. Tumors with high stromal fibrotic signatures are more likely to be associated with drug resistance and eventual relapse. Identifying the molecular underpinnings for such multidirectional crosstalk among the various normal and neoplastic cell types in the TME may provide new targets and novel opportunities for therapeutic intervention. This review highlights recent concepts regarding the complexity of CAF biology in cholangiocarcinoma, a highly desmoplastic cancer. The discussion focuses on CAF heterogeneity, functionality in drug resistance, contributions to a progressively fibrotic tumor stroma, the involved signaling pathways and the participating genes.

## 1. Introduction

**Figure 1:**
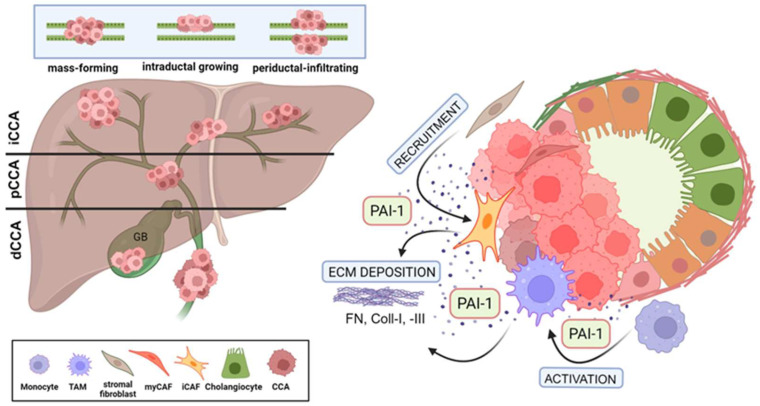
Multifunctional effects of PAI-1 on desmoplasia formation in CCA. (**A**) Cholangiocarcinoma (CCA) presents as intrahepatic (iCCA) or extrahepatic as perihilar (pCCA) or distal (dCCA) disease. Tumors spread as mass-forming, intraductal growing, and periductal-infiltating types. (**B**) PAI-1 secreted by CCA tumors (intrahepatic [iCCA] illustrated here) activates and recruits stromal cells, enriching the TME with CAFs and TAMs which then further elevate local PAI-1 concentration and, through extensive ECM deposition, support the increasing desmoplastic nature of the CCA TME. (Created by BioRender.com).

Ductular cancers are heterogenous, aggressive malignancies originating in the epithelial lining of the biliary tree both within (intrahepatic) and outside (extrahepatic) the liver. Cholangiocarcinoma (CCA) is the second most common primary liver cancer globally; approximately 10% of all CCA are intrahepatic, while 90% are extrahepatic [1,2]. Although defined risk factors are known, for reasons unclear, the incidence of CCA is increasing [1,3]. An estimated 2000–8000 Americans are diagnosed with CCA each year, and the relative resistance of this tumor to chemotherapy is a major reason for an overall dismal prognosis [4]. Subtypes of CCA likely develop through distinct processes, and the associated molecular features differ depending on anatomical location (i.e., intrahepatic, perihilar or distal tumors) [2,5,6]. While most CCAs (95%) are adenocarcinomas, patients (more rarely) may present with adenosquamous, sarcomatous or clear cell cancers [4]. A recent single-cell transcriptome analysis of human CCA revealed considerable inter- and intra-tumor heterogeneity in distal cancers, and the expression landscape of this tumor subtype was very different from that of intrahepatic carcinomas [7,8]. Independent of the tissue/organ location, CCA exhibits three distinct modes of spread: mass-forming, periductal-infiltrating, and intraductal. The majority of the perihilar and extrahepatic/distal CCA grow as either periductal-infiltrating or intraductal (papillary or tubulopapillary) tumors. Malignant cholangiocytes comprising the periductal-infiltrating tumor type traverse the mucosa, invade the bile duct wall and penetrate into the serosa (i.e., the visceral peritoneum or mesothelium), resulting in peritoneal carcinomatosis. Tumors of the intraductal phenotype are confined to the mucosal layer and spread superficially through perineural invasion and lymphatic metastasis (Figure 1). Distal CCA is a particularly lethal, highly heterogenous and largely asymptomatic or poorly diagnosed malignancy resulting in the majority of affected individuals (approximately 70%) presenting at advanced stages of disease with occult metastases or complex local involvements that preclude curative resection [4,8,9]. The growth of both periductal and intraductal tumors leads to reduced bile flow or even to the complete obstruction of the bile duct (cholestasis) with subsequent jaundice, one of the most common diagnostic symptoms of ductular cancer. Extrahepatic cholestasis results in local inflammation that in turn predisposes one to complex injury and repair processes, cholangiocyte and hepatocyte proliferation, extracellular matrix (ECM) remodeling and fibrosis. Patients that undergo even “successful” resections, eventually succumb to disease progression or ductular inflammation (cholangitis) [10]. The recurrence rate following surgical excision is high, and the median overall survival is only 35 to 48 months [11,12,13]. Additional confounders contributing to poor patient outcomes include significant uncertainties in the pathophysiological determination of CCA and its precursor lesions which may further delay treatment [14]. In this regard, improvements in radiogenomic and quantitative imaging techniques may be useful in the differential diagnosis of CCA [15]. 

Unlike the uncertainties surrounding initial diagnosis, the molecular basis underlying the initiation and progression of CCA are more apparent and involve the expression and/or mutation of oncogenes, tumor suppressor genes, gain-of-function oncogenes, and genes encoding chromatin remodeling proteins and signaling intermediates [16,17]. Although CCA is relatively rare, except in Southeast Asia and northeast Thailand specifically, where etiology appears due to infection with the liver fluke *Opisthorchis viverrini* [18], the incidence and mortality rates are increasing worldwide. Mutation frequencies in key genes may be enriched in fluke-associated cancers (e.g., *PTEN*, *SMAD4*, *TP53*, *BRCA1*) or non-fluke-related tumors (e.g., *BAP1*, *IDH1*, *IDH2*) or are found in both (e.g., *APC*, *BRAF*, *BRCA1*, *BRCA2*, *KRAS*, *TGFBR2*, *PI3KR1*, *TP53*, *SMAD4*, *NRAS*). These genes encode critical cancer-associated intermediates in several important signaling pathways [2,19,20] impacting tumor progression, aggressive behavior, and drug resistance. Whole genome sequencing implicated *TP53* and *KRAS* to be among the most frequently mutated genes (53% and 26%, respectively) and were attributed to the poor prognosis cohort [17,21]. In addition, transcriptomic and epigenetic approaches successfully catalogued genetic variations with the pathologic spectrum of CCA subtypes.

## 2. The Desmoplastic Tumor Microenvironment in CCA

CCA are highly desmoplastic tumors enveloped in a dense, rather stiff meshwork of inflammatory cells, macrophages, CAFs and ECM, the latter consisting largely of collagen type-I and the matricellular proteins tenascin C and periostin [1,2]. Desmoplasia results from an increased synthesis and deposition of ECM proteins by stromal elements and, in particular, the often-prominent population of tumor-embedded myofibroblasts [22]. Molecular and functional heterogeneity among the several CAF subpopulations are major contributors to the phenotypic complexity of the mesenchymal subsets in the TME [23,24,25]. The composition of the peritumor milieu may vary among specific malignancies but generally consists of a diverse complement of highly interactive resident and recruited cell types that coexist with the growing cancer in a hypoxic, progressively fibrotic, stromal matrix [26]. CAFs and macrophages are the most abundant nonmalignant cell types in the TME, particularly in stroma-rich desmoplastic tumors [27,28]. CAFs mediate stromal remodeling by several mechanisms including an increased post-translational modification and cross linking of ECM proteins, augmented collagen synthesis and a reduced degradation resulting in increased matrix stiffness [29] (Figure 1). The origin of the CAF population is frequently uncertain and may well be multi-sourced to include periportal fibroblasts, stellate cells, fibrocytes, vascular pericytes, transitioning macrophages, mesothelial cells and mesenchymal stromal cells [30]. In pancreatic and hepatic tumors, it appears that CAFs arise from stellate cells [31,32,33], and recent single cell RNA_seq¬_ and lineage tracing approaches implicate stellate cells and portal fibroblasts as the likely predominant sources of CAFs in intrahepatic CCA [15,30,34]. Indeed, proteins expressed by portal fibroblasts (fibulin-2) and hepatic stellate cells (desmin, GFAP) mark SMA-positive fibroblasts consistent with the possibility that both give rise to CCA CAFs [35].

The desmoplastic reaction partitions into the immature, intermediate and mature categories. For patients with extrahepatic (perihilar and distal) CCA presenting with intermediate or immature desmoplastic responses, the tumors are more invasive and have higher pT and pN stages with perineural involvement and a greater incidence of neoplastic buds [36]. Kaplan–Meier plots of overall and relapse-free survival, revealed a dismal prognosis for patients with intermediate and immature desmoplastic reactions compared to those with a mature response [36]. In intrahepatic tumors in particular, increased numbers of CAFs correlate with a greater tumor size and reduced patient survival [1,37]. The co-cultivation of two different CCA cell lines with CAFs stimulated tumor cell migration and the secretion of proinflammatory cytokines [38], suggesting that a CAF-initiated pro-metastatic function may underlie such poor outcomes.

The hypoxic and hypovascularized CCA microenvironment and extensive desmoplasia furthermore constitute a physical barrier that compromises the delivery of chemotherapeutics, further complicating treatment decisions and patient management [20]. Indeed, reciprocal crosstalk between CAFs (and other immune cells in CCA) promotes tumor progression, tissue invasion and metastasis by stimulating the expression of inflammatory and pro-tumorigenic factors, immunosuppressive cytokines and the activation of stemness pathways [39], the latter likely through the 5-LO/LTB4-BLT2 axis and TGF-β signaling [30,31,32,33,34,35,36,37,38,39,40]. In other tumor types, this dense desmoplastic stroma is the consequence of coordinated phenotypic changes in several resident populations (i.e., fibroblasts, macrophages, adipocytes, endothelial cells) and is mechanistically associated with a reduction in CD36 expression [41]. It appears, therefore, that desmoplasia may not just be a tissue response to an aggressive cancer; it may also create a survival niche for tumor cells [42]. 

## 3. CAF Subtypes in Cholangiocarcinoma

There are approximately 14 different CAF phenotypes in human tumors. Two in particular, the inflammatory (iCAFs) and the myofibroblastic (myCAFs) variants, are prominent among the six subtypes of CAFs evident in CCA [17,34,39]. Although there is some phenotypic plasticity evident [43], the iCAFs and myCAFs express a restricted repertoire of biomarkers [44]. While the consensus is that iCAFs are tumor-promoting (iCAFs are associated with the growth of intrahepatic CCA) [44,45], it appears that myCAFs may be both tumor-promoting and tumor-restraining; however, the inter-conversion between these two cell types and the progressive complexity of the TME make such distinctions uncertain [44]. It is now apparent that multilevel interactions among tumor cells and CAF subsets instruct CAFs to acquire ECM remodeling abilities that contribute to the maintenance of cancer stemness and the transition to a tumor-promoting, fibrotic niche-forming phenotype. Reciprocal crosstalk among CAFs, immune cells (granulocytes, B and T lymphocytes, natural killer cells and dendritic cells), tumor-associated macrophages, endothelial elements and tumor stem cells promote cellular plasticity and tumor progression, the maintenance of cancer cell stemness, metabolic reprogramming, chemoresistance, ECM remodeling and metastasis within a dynamic stromal environment [44,46,47,48,49,50,51,52]. Indeed, the stratification of tumor subtypes based on ECM signatures can predict cancer progression; in fact, the pro-fibrotic “matrisome” is associated with invasive signaling in squamous cell lung carcinoma with poor prognosis [53]. A specific complement of ECM remodeling genes not only portends an increased risk of squamous cell lung cancer risk but a poor outcome as well. Clarifying the critical pathways in the CAF-tumor interacting network, may provide new venues for therapeutic intervention [47,48,54,55].

## 4. Signaling Pathways Involved in CCA Desmoplasia 

**Figure 2:**
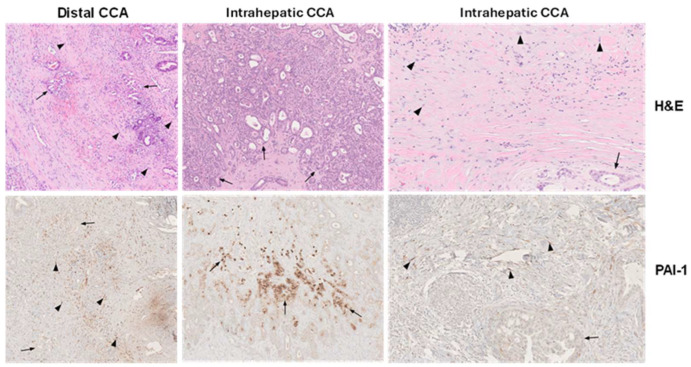
Immunohistochemical localization of PAI-1 in human CCA. Hematoxylin and eosin (H&E)-stained distal and intrahepatic human CCA depict tumor cells (arrows) and fibroblasts (arrowheads) within the desmoplastic CCA microenvironment (top panels) (×40) with the corresponding SERPINE1 (PAI-1) immunolocalization (bottom panels) in serial sections. Abundant fibroblasts in distal CCA are PAI-1-positive, whereas tumor cells are largely negative (left bottom panel (×40). In intrahepatic CCA, prominent focal PAI-1 immunoreactivity is evident in some CCA tumoral glands (middle bottom panel) (×40) and intensely in stromal fibroblasts (right bottom panel) (×40) [66]. The normal liver tissue, including portal tracts and lobules, is PAI-1-negative.

The development of a CAF-enriched TME and eventual tumor progression involves engagement of several signaling systems including the canonical (SMAD) and noncanonical (non-SMAD) arms of the TGF-β network [25,50,56,57,58,59,60,61,62]. The TGF-β pathway is a major contributor to the development of CCA and activates both SMAD-dependent and independent genomic programs, resulting in the maintenance of cancer stemness, acquisition of a plastic phenotype and an intense desmoplastic reaction [63]. In intrahepatic CCA, the TGF-β-specific gene signature includes inflammatory mediators and the potent profibrotic genes *SERPINE1* (which encodes plasminogen activator inhibitor-1; PAI-1) and *CCN2* [64]. PAI-1 is particularly relevant as this serine protease inhibitor is expressed by tumor cells and endothelial elements in the TME but also, in particular, by the CAF population [49,65,66,67,68] (Figure 1 and Figure 2). The mechanisms whereby PAI-1 contributes to the progression of malignancy may vary per tumor type and TME complexity. CAF-derived PAI-1 stimulates not only ECM deposition and accumulation but also cancer cell invasion and macrophage migration, resistance to chemotherapy, the attenuation of FasL-mediated apoptosis, tumor cell locomotion and amoeboid motility and the promotion of lymphatic metastases [49,67,69]. Despite the appreciable evidence for a significant and multifaceted contribution of PAI-1 in promoting the development of an aggressive TME, the potential role for PAI-1 in CCA is largely unknown.

Pathways activated by TGF-β1 in the TME that contribute to tumor progression and aggressive behavior also include those involving p53, the Hippo effectors YAP (*yes*-associated protein) and TAZ (transcriptional coactivator with PDZ-binding motif) and AKT [57,70,71,72]. p53 is specifically notable as p53 expression and mutational status are both significantly increased in CCA [73,74,75]. This has clinical implications as p53 is a critical co-activator of *SERPINE1* transcription in the genomic profibrotic and cellular senescence programs [70,76,77,78,79,80]. Such transcriptional outcomes, moreover, are not restricted to TAp53 as TAp63, TAp73a and the N-terminal truncated function-compromised isoform of TAp63 (ΔNp63) also transactivate the *SERPINE1* gene [81,82,83]. The Kaplan–Meier analysis of clinical data from patients with intrahepatic CCA, in fact, established an elevated expression of the dual TAD/proline domain-truncated Δ133p53 isoform and high Δ133p53/TAp53 ratios as prognostic factors for poor overall survival [84].

Similarly, TAZ is also a key intermediate in TGF-β1-induced fibrogenesis, and engineered TAZ overexpression is critical to G_2_/M arrest and the initiation of a profibrotic program [71]. Fundamental in this response appears to be the requirement for TAZ participation in the TGF-β1-stimulated expression of the SMAD target gene *SERPINE1* [71,85,86,87], and a similar involvement of YAP in TGF-β1-induced PAI-1 expression is evident in lung tumor cells [88]. KEGG analysis confirmed, moreover, the convergence of the TGF-β, p53 and Hippo signaling pathways in the transcription of the fibrosis-inducing connective tissue growth factor (*CCN2*) and *SERPINE1* genes [89]. YAP knockdown effectively reduces levels of both CCN2 and PAI-1, while the introduction of the constitutively active YAP^S127A^ construct increases PAI-1 levels in immortalized cell lines [90]. It is apparent, moreover, that YAP and TAZ integrate bidirectional responses between tumor and stromal cells functioning as signaling hubs, perhaps in response to increasing cellular tensile forces as well as the changing mechanical properties (i.e., progressive stiffening) of the TME [91,92,93].

The serine/threonine protein kinase AKT is a critical downstream transducer of the PI3K pathway and its cellular functions [72]. The AKT1, 2 and 3 isoforms appear to have differential, non-redundant and opposing effects on tumor cell and fibroblast motility, although, collectively, AKT exerts significant effects on the development of the peritumor stroma [72,94]. Since the kinase domains of AKT1, 2 and 3 are highly conserved and many of the target substrates may well be shared, sequence-related protein–protein interactions and/or the relative abundance of the three AKT isoforms may determine the impact on the cellular motile program [95,96]. Recent findings, moreover, link the overexpression of PAI-1 to the activation of the PI3K pathway, the phosphorylation of AKT^Thr308^ and increased tumor cell survival [97]. Indeed, higher levels of PAI-1 are evident in several metastatic tumors and associated with shorter overall survival and poor prognosis [97]. The genetically engineered overexpression, or exogenous addition, of PAI-1 stimulates AKT phosphorylation, and pharmacologic inhibitors of PAI-1 activity significantly attenuate pAKT levels [98]. While the mechanisms whereby PAI-1 targets and activates the PI3K/AKT pathway are uncertain, the involvement of the LDL receptor-related protein 1 (LRP1) appears likely [99]. The binding of PAI-1 to cell surface LRP1 stimulates Jak/Stat-dependent motility in human melanoma and breast cancer cells [100] as well as migration and tissue invasion in esophageal squamous cell carcinoma through the activation of AKT and ERK1/2 signaling pathways [101]. PAI-1/LRP1 interaction can be blocked with the PAI-1-specific inhibitor TM5275 [102] which has anti-proliferative effects in ovarian cancer cells [103]. PAI-1/LRP1 signaling, furthermore, may well hijack two important receptor systems in CCA, PDGFRβ and DDR1. PDGFRβ is highly expressed in the desmoplastic TME in CCA and connects PAI-1/LRP1 to the PI3K/AKT and Stat pathways [104,105,106,107]. PDGFRβ activation contributes to drug resistance in CCA [108], and DDR1 is a major cell surface receptor for collagen-1 signaling through the PI3K/AKT/mTOR and MAPK networks [109]. DDR1 is upregulated in highly fibrotic HCC and associates with poor prognosis, particularly in extrahepatic CCA [110]. Cell surface DDR1 interacts with LRP1, which regulates the endocytic clearance of DDR1, thereby stimulating cellular proliferation and cell-cycle progression [111]. LRP1 therefore appears to be a potent entry portal to several important signaling pathways potentially activated by PAI-1 to provide pro-survival signals to CCA cells.

The available data further suggest, moreover, that PAI-1 is a key factor in the TGF-β1-driven transformation of fibroblasts to a myofibroblastic phenotype. Indeed, flexor tenocyte myofibroblast differentiation in response to TGF-β1 is significantly attenuated in *SERPINE1*-deficient mice [112], and incubation with SK-216, a pharmacologic inhibitor of PAI-1 activity, effectively blunts the TGF-β1-induced transition of MRC-5 fibroblasts to myofibroblasts as well as the epithelial-to-mesenchymal conversion in TGF-β1-treated A549 lung epithelial cells [113]. PAI-1 binding to urokinase also induces corneal myofibroblast differentiation on a vitronectin substrate [114], while the treatment of dorsal skin wounds in FVB/NJ mice with the small molecule PAI-1 antagonist Tiplaxtinin dramatically reduced wound closure and re-epithelialization [115]. Tiplaxtinin effectively attenuates keratinocyte migration and myofibroblastic differentiation. Collectively, these data suggest that, regardless of the specific TGF-β1 pathway activated (e.g., p53, YAP/TAZ, PI3K/AKT), PAI-1 regulates myofibroblast differentiation both in vitro and in vivo and may well do so in the CCA TME.

## 5. Multifunctional Impact of PAI-1 on Cholangiocarcinoma Progression 

**Figure 3:**
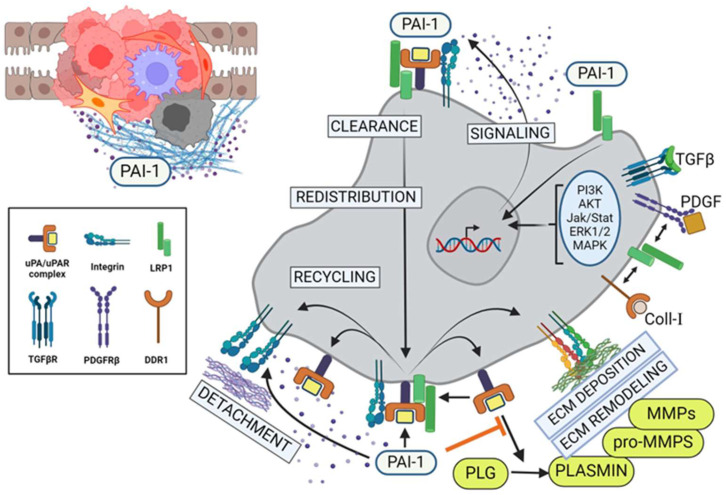
PAI-1 alters TME and regulates tumor cell migration and invasion. PAI-1 modulates pericellular proteolysis by titrating the activity of uPA-dependent plasmin generation and subsequent upregulation of matrix metalloproteinases (MMPs), leading to extensive ECM remodeling. In parallel, the binding of pericellular PAI-1 directly to the endocytic receptor, LRP1, results in activation of the Jak/Stat1 signaling pathway and de novo synthesis of PAI-1. Consequentially, this elevated stromal PAI-1 increasingly binds to tertiary cell surface uPA/uPAR/integrin complexes, leading to inactivation of those integrins and their detachment from the ECM, resulting in local cell detachment. Upon recruitment of LRP1, endocytic clearance of these inactive complexes leads to lysosomal degradation of the uPA/PAI-1, subcellular redistribution and recycling of the receptors to the leading edge of the tumor cells, promoting locally reattachment (integrins), transient pericellular proteolysis (uPAR) and further cycles of PAI-1-driven receptor internalization and recycling (LRP) in support of increased cell motility and invasion. PLG = plasminogen; MMP = matrix metalloproteinase. (Created with BioRender.com).

Certain CAF genes are markedly upregulated in response to progressive hypoxia including that encoding PAI-1, which promotes aggressive behavior in various malignancies [49,67,116,117,118,119]. Unsupervised clustering identified a slate of differentially expressed and hub genes in CCA subtypes that may have prognostic implications [7,120,121]. Comparative BeadChip array analyses of normal biliary epithelial cells and differentiated vs undifferentiated CCA cells disclosed three distinct gene clusters [4]. Cluster II, which represents genes upregulated in differentiated CCA and downregulated in the normal epithelium, includes the matricellular proteins SPARC and PAI-1 and three collagen variants including COL5A1, COL1A1 and COL6A3 [4]. It is interesting that PAI-1 is also the most highly expressed gene in bile duct ligation-induced cholestatic injury [122], suggesting a critical role in both biliary malignancies and tissue repair. PAI-1 limits plasmin generation and, as such, promotes stromal invasion (at least by transplanted tumors and angiogenic endothelial cells) by preserving a matrix “scaffold” permissive for migration [123] (Figure 3). The function of this serine protease inhibitor, however, may be more complex and not just restricted to its anti-proteolytic properties as recent findings suggest a more general role in the regulation of cell-to-substrate adhesion [49,124,125,126,127,128,129,130].

**Figure 4:**
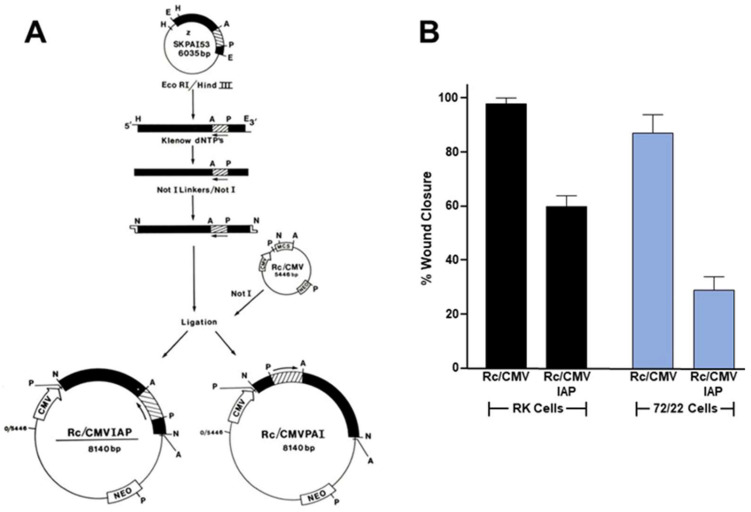
PAI-1 knockdown attenuates cell motility. Established rat 72/22 hepatic epithelial cells, derived from the liver of diethylnitrosamine-treated rats express cytokeratin (CK) proteins typical of transitional ductular hepatocytes (i.e., CKs 8 [55 kDa], 18 [49 kDa], 19 [40 kDa]) as well as significant levels of the 57 kDa homopolymer intermediate filament (IF) protein vimentin [154]. Moreover, 72/22 cells are CK19^+^/AFP^−^/ALB^−^, supporting a ductular origin. Transition of hepatocytes to bile duct epithelia involves the synthesis of the hepatocyte-characteristic CKs 8 and 18 followed by CK19 [155]; expression of CK7 appears to be the last event in ductular differentiation. Collectively, these previous findings support the identification of 72/22 cells as an early stage ductular phenotype. A 52 kDa protein (p52) co-isolated with the 72/22 cytoskeletal fraction [138,154,156]. Although p52 migrated just below CK8 on denaturing one-dimensional gels, as did degradation products of CK8, this protein is unrelated to either CK8 or vimentin as it resolved as a complex of considerably more basic species in two-dimensional separations [156]. Differential subcellular extraction and imaging procedures, moreover, indicated that p52 selectively localized to the undersurface extracellular compartment of 72/22 cells [156]. Subsequent proteolytic fragment mapping and combined two-dimensional electrophoresis/Western analyses revealed p52 to be identical to the extracellular matrix-associated 52 kDa type-1 inhibitor of plasminogen activator (PAI-1) [138,156] also known as SERPINE1 (serine protease inhibitor, clade E type-1). To assess the role of PAI-1 in epithelial cell migration, PAI-1 sense and anti-sense (IAP) expression vectors [157] were constructed and transfected into rat keratinocytes (RK cells) and cholangiocarcinoma (72/22) cells. PAI-1 expression knockdown by the Rc/CMVIAP construct was confirmed by Western blotting [145]. After selection in G418, transfectants were allowed to grow to confluence, the monolayers scraped injured and the % wound closure determined as described previously [145,150].

Indeed, PAI-1 can displace cells from various ECM substrates (e.g., vitronectin, fibronectin, collagen-1, laminin-5) by initiating uPA receptor (uPAR) deactivation and LRP1-mediated clearance of matrix-engaged integrins [131,132,133,134]. In colorectal cancer, elevated levels of PAI-1 are significantly correlated with perineural invasion, increased metastasis and the expression of EMT-associated genes (e.g., PDGFR-B, r = 0.71) and are predictive of lower overall survival [135]. uPA and uPAR are also upregulated in subserosal/serosal tumors compared to smaller tumors in the mucosa and submucosa [136]. Interestingly, high expression ratios of PAI-1 compared to uPAR (r = 0.490) and receptor-bound uPA (r = 0.469) strongly correlate with tumor-size, suggesting a predominant role for high PAI-1 expression in tumor growth rather than just the control of plasmin generation. These data suggest an intriguing mechanism by which PAI-1 regulates the presence and location of surface adhesion receptors (integrins), protease receptors (uPAR) and endocytic receptors (LRP1) to support directed cell migration and tissue invasion (Figure 3). Alternatively, PAI-1 may directly inhibit αv integrin–vitronectin attachment by blocking accessibility to the RGD sequence located proximal to the uPAR and PAI-1 binding sites [130,134,137]. The endocytosis of uPAR-associated uPA/PAI-1 complexes, moreover, promotes uPAR recycling and therefore vitronectin-dependent cell movement [137]. PAI-1 in fact accumulates specifically in the cellular undersurface region [134,137,138,139,140] where it is well positioned to modulate integrin–ECM or uPA/uPAR-ECM interactions as well as stromal proteolysis [141,142]. Since PAI-1 is deposited into migration tracks [131,143,144,145], changes in PAI-1 expression may influence motile behavior by modulating either uPA-dependent pericellular proteolysis or cell-to-matrix adhesion [130,131,134,138,146,147,148,149]. The anti-sense downregulation of PAI-1 synthesis or the use of PAI-1 function-blocking antibodies in fact inhibits basal as well as growth factor-stimulated cell motility in two- and three-dimensional culture models [128,150,151,152,153]. Consistent with findings implicating PAI-1 in tumor aggressiveness, cell migration and metastasis [49,66], the anti-sense down regulation of PAI-1 in CCA cells effectively attenuated scrape wound-stimulated migration (Figure 4), suggesting that targeting this SERPIN in the TME, regardless of the cell source, may have therapeutic utility in the management of metastatic disease.

## 6. Conclusions

The various subpopulations of CAFs within the TME are in close proximity to, and communicate with, the growing tumor mass and other resident mesenchymal elements. In neoplasms that present with an intense accumulation of fibrous connective tissue (e.g., stroma-rich desmoplastic tumors), CAFs are often the prominent cancer-associated mesenchymal cell type where their presence and abundance signal a poor prognosis. CAFs remodel the stromal matrix into a dense, fibrotic structure while secreting growth factors and cytokines that promote the acquisition of aggressive growth characteristics. Tumors with high stromal fibrotic signatures are more likely to be associated with drug resistance and eventual relapse. Various CAF subpopulations support tumor progression and cancer cell phenotypic transitions largely through paracrine signaling by a diverse complement of secreted growth factors and cytokines [24,49]. While CAFs may promote tumor growth and survival through reduced sensitivity to apoptotic stimuli, these responses are largely context-dependent and the underlying mechanisms difficult to dissect due to CAF diversity and origin, involved tumor type and the spectrum of CAF functions. It is clear, however, that a cooperative dialogue between CAFs and cancer stem cells promotes cellular plasticity and tumor progression, drug resistance, the maintenance of cancer cell stemness, ECM remodeling and metastasis. 

The contrasting effects of CAFs on tumor cell populations in vivo when compared to in vitro systems highlight the importance of assessing CAF function in animal models [45]. Hepatic stellate cells appear to be the main CAF source in CCA, and two of the most prominent CAFs (iCAFs and myCAFs) have distinct roles in fibroblast activation. While stellate cells differentiate into iCAFs, which subsequently give rise to myCAFs, it is likely that both states are transient and CAFs shuttle between the two phenotypes [45]. Clarifying the critical pathways in the CAF–tumor interacting network may provide new venues for therapeutic intervention. The mounting evidence of CAF heterogeneity and pro-tumorigenic functional complexity, including their ability to continuously modify the stromal structure of the TME through several cooperative pathways, suggests that the development of targeted therapies will require a multidimensional approach. Exploring new strategies, focusing on crosstalk among the various normal and neoplastic cell types present in the TME, including the use of specific molecular and pharmacologic approaches to inhibit the function of key tumor progression genes (e.g., *SERPINE1*), may have clinical utility e.g., [118,158,159,160,161]. Indeed, the selective targeting of PAI-1 with several small molecule inhibitors increased the susceptibility of chronic myeloid leukemia stem cells (CML-LSCs) to imatinib chemotherapy in a mouse model [162]. The combined treatment of CML-LSC-bearing mice with PAI-1 inhibitors and imatinib resulted in the depletion of tumor cells in the bone marrow, prevented recurrences and prolonged survival times. While no such preclinical data are available for assessment of PAI-1 blockade in CCA, it is tempting to speculate that such combination modalities may have utility in the management of patients with highly desmoplastic tumors. 

PAI-1 is highly upregulated (9-fold) in cholestatic injury, reflecting stellate cell activation and collagen synthesis with subsequent hepatic fibrosis, in response to bile duct ligation [122,163]. Genetic *SERPINE1* deficiency mitigates disease progression, highlighting the role of this protease inhibitor in liver fibrosis [122,163,164]. While the mechanism is uncertain, levels of tissue-type plasminogen activators, matrix metalloproteinase-9 and the anti-fibrotic hepatocyte growth factor are significantly increased in PAI-1-null mice, and the oral administration of the small molecule PAI-1 inhibitor TM5275 attenuates hepatic fibrosis [164,165]. PAI-1 upregulation is common in various liver diseases, although targeting PAI-1 expression and/or function remains problematic. Uncertainties regarding PAI-1 attenuation on hepatitis virus C replication, the instability and short half-life of the PAI-1 protein, the hemostatic consequences of PAI-1 disruption and the role of PAI-1 in adaptive and pathophysiological wound repair remain important challenges in the design of PAI-1-based hepatic therapeutic strategies [166,167,168].
